# Evolving Strategies for Cancer and Autoimmunity: Back to the Future

**DOI:** 10.3389/fimmu.2014.00154

**Published:** 2014-04-14

**Authors:** Peter J. L. Lane, Fiona M. McConnell, Graham Anderson, Maher G. Nawaf, Fabrina M. Gaspal, David R. Withers

**Affiliations:** ^1^MRC Centre for immune Regulation, Birmingham Medical School, Birmingham, UK

**Keywords:** CD4 T cell, autoimmunity, tolerance mechanisms, cancer, regulation, memory, keyword

## Abstract

Although current thinking has focused on genetic variation between individuals and environmental influences as underpinning susceptibility to both autoimmunity and cancer, an alternative view is that human susceptibility to these diseases is a consequence of the way the immune system evolved. It is important to remember that the immunological genes that we inherit and the systems that they control were shaped by the drive for reproductive success rather than for individual survival. It is our view that human susceptibility to autoimmunity and cancer is the evolutionarily acceptable side effect of the immune adaptations that evolved in early placental mammals to accommodate a fundamental change in reproductive strategy. Studies of immune function in mammals show that high affinity antibodies and CD4 memory, along with its regulation, co-evolved with placentation. By dissection of the immunologically active genes and proteins that evolved to regulate this step change in the mammalian immune system, clues have emerged that may reveal ways of de-tuning both effector and regulatory arms of the immune system to abrogate autoimmune responses whilst preserving protection against infection. Paradoxically, it appears that such a detuned and deregulated immune system is much better equipped to mount anti-tumor immune responses against cancers.

## Introduction

In our society today, cancer and autoimmunity are major causes of suffering and death, and a huge financial burden on health services worldwide. The strongest genetic link with autoimmunity is to major histocompatibility (MHC) class II genes, implicating CD4 T cells in autoimmune pathogenesis. Less obviously, CD4 T cells are also implicated in defective immunity to tumors, as CD4 regulatory T cells (Tregs) limit effector responses to tumor antigens.

Our studies have centered on CD4 immunity and its regulation, and have been informed by the striking observation that the key features of the CD4 immune system – high affinity antibody responses, memory, and CD4 regulation – co-evolved with placentation in mammals (1–3). A simple comparison of the numbers of species in different mammalian groups – monotremes, 2; marsupials, ~400; placentals, ~5000 – illustrates the reproductive advantage conferred by placentation. The contribution of the immune system to this advantage is threefold: the bringing of unborn young to immunocompetence at birth; protection after birth by maternal transfer of high affinity IgG; and reduction in exposure of offspring to disease epidemics due to memory responses in the community, the latter two being CD4 T cell dependent functions. Note that the pressure on the immune defenses due to the physical frailty of the placental newborn is enormous, as demonstrated by the fact that even with the immune protections described, mortality is exponentially higher in infants than in adults (4). The high potency of effector immune responses demanded by placentation carries high risk of pathological autoimmunity, which has been substantially addressed by the co-evolution of T regulatory mechanisms. But because Darwinian selection favors reproductive success rather than individual survival, the protection of the developing fetus takes precedence over the risk of personal suffering and even death in post-reproductive adults. And we believe that the less than complete limitation of autoimmunity by T regulation already carries its own risk, also consequent on the stringent necessity of reproductive success. Before placentation, protection of the self from effector responses required tolerance to tissue specific self-antigens; the evolution of placentation required this tolerance to extend to placenta- and fetus-specific antigens as well. Additionally, we think that the intermittent nature of pregnancy fosters the selection of dominant forms of tolerance (see Evolution of the Placenta – A New Organ). Our view is supported by two lines of evidence: – the wide expression of fetus- and placenta-specific antigens by human cancers in both males and females (5), reflecting selection of cancer cells that can gain advantage from the immune regulation protecting the developing embryo and fetally derived placenta; – recent identification of memory Tregs induced specifically by fetally derived antigens (6). Although deletional tolerance no doubt also operates to maintain tolerance to fetally derived antigens, the particular advantage of dominant tolerance to fetal antigens expressed in the placental trophoblast is that it could also confer bystander tolerance to fetal alloantigens at the feto-maternal interface, to which there is no opportunity for maternal thymic tolerance, particularly during first time pregnancies before there is priming to induce Tregs specific for alloantigens (6).

**Figure 1:**
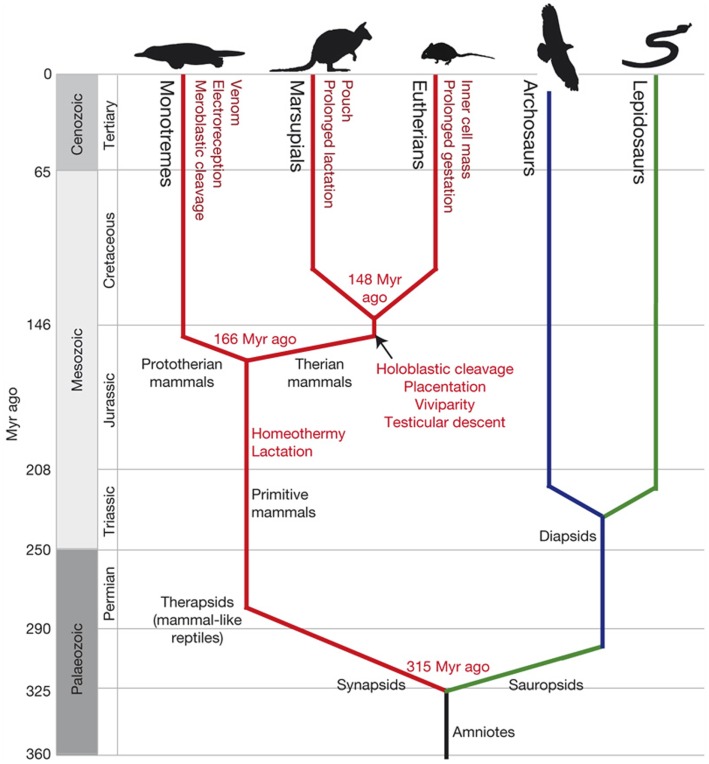
**Emergence of traits along the mammalian lineage**. Amniotes split into the sauropsids (leading to birds and reptiles) and synapsids (leading to mammal-like reptiles). These small early mammals developed hair, homeothermy, and lactation (red lines). Monotremes diverged from the therian mammal lineage ~166 Myr ago and developed a unique suit of character (dark-red text). Therian mammals with common characters split into marsupials and eutherians around 148 Myr ago (dark-red text). Geological eras and periods with relative times (million years ago) are indicated on the left. Mammal lineages are in red; diapsid reptiles, shown as archosaurs (birds, crocodilians, and dinosaurs), are in blue; and lepidosaurs (snakes, lizards, and relatives) are in green.

In this article, we start by reviewing the fundamental changes that occurred in mammals over the last 200 million years [see Figure 1 Ref. (7)] and that form the context of the sequential genetic changes that enabled new immunological structures and functions to evolve. We then review the evidence that the resulting “modern” mammalian immune system can be detuned to give a minimal essential immune system for health: a system that, without compromising immunity to infection, can both abrogate pathogenic CD4 driven autoimmune responses and augment anti-tumor immunity.

## Brief History of Mammals from an Immunological Perspective

Comparative genomics and the fossil record have been used as a timeline for the evolution of mammals from the common reptilian ancestor [Figure 1 Ref. (7)]. This timeline highlights the major physiological changes that emerged: the evolution of homeothermy, of hair (insulation), and of lactation (an effective strategy to nourish homeothermic offspring). All mammals, including the egg laying monotremes, have these characteristics, which distinguish them from the cold-blooded common ancestor they share with reptiles and birds.

### Origins of lymph nodes

The development of these characteristic mammalian attributes was accompanied by changes in the immune system, both anatomically and functionally. The most striking gross anatomical change is the emergence of intra-lymphatic lymphoid aggregates (the ancestors of lymph nodes) in the common mammalian ancestor. All jawed vertebrates have a spleen and lymphoid aggregates in the mucosal associated lymphoid tissues, but only mammals have lymphoid structures that are intra-lymphatic as opposed to lymphatic-associated. In chickens, lymphoid aggregates are found in the wall but not the lumen of lymphatics (8), which is a similar arrangement to the so called isolated lymphoid follicles (ILFs) found at mucosal sites in all vertebrates, and associated with but not inside lymphatics (e.g., the lacteals in the gut). In contrast, vascularized intra-lymphatic ILF-like structures are found in both of the extant monotremes (9, 10). Why did these intra-lymphatic structures, which are the precursors of mammalian lymph nodes, evolve? Possible explanations in our view are linked to the evolution of homeothermy in mammals, with the consequent increased demand for nutrients. The mucosal system of mammals has a much more extensive surface area for absorption of food than reptiles, and consequently has increased exposure to bacteria, both commensal and non-commensal, in the gut. One view therefore, is that these intra-lymphatic lymphoid structures evolved in the mesentery of the gut as a “firewall” to block the entry of gut bacteria into the systemic circulation (11). In any case, comparative genomics supports the view that intra-lymphatic lymphoid structures evolved in the common mammalian ancestor, as the L-selectin gene (SELL) first appears in monotremes (www.ensembl.org). This molecule occurs on lymphocytes and directs their exit from blood vessels into lymphatics (as lymph nodes are essentially intra-lymphatic structures), and is not present in reptiles or birds, which however do have genes for the inflammatory selectins present on endothelium, E-(SELE), and P-selectin (SELP). SELE and SELP are chromosomally co-located in mammals with L-selectin, indicating that SELL is likely to have arisen by gene duplication in the common mammalian ancestor.

Microscopically, the ILFs found inside the lymphatics of monotremes contain B cell germinal center (GC)-like structures with follicular CD4 T cells (10), but they do not contribute to affinity maturation of the B cell response or to memory; secondary antibody responses in monotremes are very similar to primary ones (2). In the absence of a link to affinity maturation, it seems probable that these GC-like inclusions mediated the diversification of the B cell repertoire, after the fashion of the GC-like structures seen to develop in the Bursa of Fabricius in chickens, where the B cell repertoire is diversified by activation induced cytosine deaminase (AID)-dependent gene conversion of immunoglobulin variable region genes (12). In placental animals like sheep, primary diversification of the B cell repertoire through gene conversion and somatic hypermutation also occurs in the gut associated lymphoid tissues (13), so it is quite plausible that this is the function first appearing inside intra-lymphatic nodules in monotremes. Orthologs of IgE and IgG also evolved in the common mammalian ancestor; in monotremes (14), unlike placental animals, switched immunoglobulin isotypes are produced in primary immune responses, and it is possible that the sites of AID-dependent class switching, to not only IgE and IgG, but also IgA, are the intra-lymphatic ILFs.

### IgA control of anti-inflammatory and responses to gut microbiota

IgA is the most abundant immunoglobulin, and secreted IgA is the main immunoglobulin at mucosal surfaces. Orthologs of IgA are present in reptiles, birds, and mammals but not amphibians and fish, indicating that this immunoglobulin class evolved in the common amniotic ancestor of land animals. The evolution of the amniotic egg was critical to the capacity of vertebrates to colonize non-aquatic habitats, as it freed their reproduction from the dependency on access to water retained by amphibian species. However, terrestrial habitats brought new challenges in the form of different food sources and different microbiota colonizing the gut. In this context, the anti-inflammatory properties of IgA are likely to have been crucial. Tsuji et al. (15) present several pieces of evidence pointing to the importance of IgA in maintaining a normal gut flora. AID-deficient mice developed abnormal gut flora, which was associated with impaired switching to IgA in ILFs in the mucosa. This IgA-dependent class switching was shown to be T cell independent, but dependent on AID expression in ILF GC-like structures.

### Origin of CD4 immunity

Modern mammals share a 450-Myr-old common ancestor with cartilaginous fish, and indeed all jawed vertebrates have RAG-dependent adaptive immune systems. However, recent sequencing of the elephant shark genome has revealed no evidence for the CD4 gene, and indeed no evidence of either FoxP3, the gene linked to evolution of CD4 regulation (16), or ROR-gamma (RORc), the transcription factor required for the development of Th17 CD4 T cells (17). This suggests that CD4 T cells were not part of the ancestral RAG-dependent adaptive immune system, but evolved later. Recent data have implicated both CD4 Th17 cells and Tregs in IgA immunity.

### FoxP3^+^ and Th17 CD4 T cells are involved in IgA production in the gut

The FoxP3 gene is highly conserved in all placental mammals (www.ensembl.org). Given that members of the FoxP gene family are consistently highly conserved across all animal groups, this would not be surprising, except that by comparison, FoxP3 orthologs in marsupials, marsupials, reptiles, monotremes, fish, and amphibians show much reduced conservation. The step gain in conservation of FoxP3 in placental mammals is consistent with a gain of function specific to placentation (18). Similarly, IL17a, the principle IL17 cytokine secreted by Th17 T cells, is exclusive to mammalian genomes, and therefore evolved in the common mammalian ancestor.

The conventional view of FoxP3^+^ Tregs is that their primary role is to suppress or modulate CD4 dependent immune responses (19), but there is evidence that they also function in the T cell dependent switching to IgA in the gut: Foxp3 expressing Tregs were potent inducers of IgA after transfer in T cell deficient mice (20); Tregs were found to be important mediators of induction of IgA to flagellin, a molecule common to commensal and pathogenic bacteria (21). The latter result raised the very interesting idea that a key function of Tregs is to promote the generation of IgA antibodies to commensal bacteria in the gut, so maintaining homeostasis of gut microbiota and preventing inappropriate inflammatory immune responses. A possible role for the ancestral FoxP3-dependent Tregs could therefore be to moderate immune responses to commensal bacteria by effectively providing help for B cells.

This suggestion is somewhat controversial, as recent studies have implicated Th17 cells rather than Tregs in the induction of IgA in the gut (22, 23). Cao et al. implicated the mammalian specific cytokine IL17a in the production of IgA to flagellin. The available data are however not necessarily contradictory, as in humans at least a significant fraction of Tregs isolated from mucosal surfaces co-express FoxP3 and RORγt, the key Th17 transcription factor (24), in addition to producing IL17a. A scenario compatible with the evidence is that there was co-evolution of Th17 and FoxP3^+^ T cells in the common mammalian ancestor, at least in part to promote IgA antibody production in the gut, and in any case to act synergistically to promote integrity at mucosal surfaces.

### Evolution of high affinity antibodies and memory in the common placental ancestor

As discussed earlier (see “Origins of Lymph Nodes” above), despite having intra-lymphatic lymphoid follicles containing GCs, monotremes do not make high affinity antibody responses or demonstrate CD4 memory (2, 9, 10). They do not have true lymph nodes with segregated B and T cell areas, but these structures evolved in the common placental ancestor; all marsupials and placental mammals have lymph nodes, and demonstrate the capacity to make high affinity antibodies and memory (1, 3, 25, 26). These advances are linked to the evolution of new genes, notably the lymphotoxin βreceptor (LTβR) and its ligands, which are not only essential for lymph node development but also for making high affinity antibodies and memory.

### Lymphoid tissue inducer cells: Rorγ dependent, and linked with both lymph node and CD4 memory development

Reina Mebius was the first to characterize murine lymphoid tissue inducer cells (LTi) (27). Their function in the development of lymphoid tissues was revealed when it was found that mice deficient in the orphan retinoic acid receptor gamma (RORγ) lacked both this CD4^+^CD3^−^ LTi population and lymph nodes (28, 29). These studies link LTi unequivocally with the development of lymph nodes, cryptopatches, and ILFs through their expression of the tumor necrosis superfamily members (TNFSF) for the lymphotoxin beta receptor (LTβR), LTα_1_β_2_ (30), and TRANCE (31); more recent studies have also shown that LTi are rich sources of the cytokine, interleukin 22 (IL22) (32), which is associated with the promotion of defenses at epithelial sites (33–35). This would permit the LTi a function in the promotion of innate immunity that is distinct from the induction of lymphoid tissues.

Additionally, our work has shown that LTi persist in adult lymphoid tissues in both mouse (36) and man (37), but are distinguished from the neonatal population by their expression of high levels of OX40-ligand (OX40L)(TNFSF4) (36, 37) and in mouse, CD30L(TNFSF8) (36). Our studies have found that CD4 T cell memory function is highly dependent on signaling through both OX40 and CD30 (38), suggesting additional roles for LTi in the mediation of adaptive CD4 dependent immune responses.

### Importance of the immune contribution to placentation

It is difficult to refute the significance of the co-evolution of high affinity antibodies and lymph nodes with placentation. Zinkernagel (39) takes the view that memory and high affinity antibodies are chiefly relevant because they protect offspring via maternal transfer of high affinity antibodies, and the way in which immunological functions have evolved during placentation broadly supports his perception. We know that orthologs of IgG first appeared in the common mammalian ancestor because monotremes have them (14). The neonatal Fc receptor (FCGRT), however, which is the gene that enables the crucial transfer of IgG from mother to offspring as well prolonging the beneficial effects of IgG by increasing its half-life (40), is only present in marsupial and placental genomes. Studies of human infant mortality to most common infections show an exponential decline with the age (4) with exception of the first year of life where transfer of maternal IgG plays a crucial role in infant survival through protective immunity. Indeed in many placental animals failure to transfer maternal IgG is fatal. Comparative genomics therefore supports the idea that the development of the capacity to make high affinity antibodies and transfer them to offspring is an integral component of the evolution of a fundamental change in reproductive strategy.

### Co-evolution of high affinity antibodies with FoxP3-dependent regulation

In placentals, GCs in B follicles are the locations where T cell dependent B cell selection drives the generation of high affinity antibodies (41), but there must exist mechanisms to edit self-reactive GC B cells that acquire self-reactivity. This can occur because self-proteins can be inadvertently conjugated to foreign proteins (e.g., apoptotic virally infected cells), and therefore GC B cells that acquire self-reactivity by chance have the capacity to get help from GC follicular T helper cells. Indeed, it is very common in viral infections in humans to get transient low affinity autoantibody production, but this does not usually go on to generate high affinity class switched autoantibodies.

Recent studies have shown that Tregs are also present in normal GC (42, 43) and the fact that high affinity IgG autoantibodies to a wide variety of tissue-restricted antigens are found in FoxP3-deficient mice indicates that Tregs must be pivotal in preventing the generation of these autoantibodies. Although it is by no means clear how Tregs in GC prevent autoantibodies being generated, there is better evidence for how self-specific Tregs are selected. Recent work has shown the critical role of the thymic medulla in the selection of thymic derived regulatory but not conventional T cells (44). The gene AIRE, expressed in the thymic medulla, controls the expression of many tissue-restricted antigens (45, 46). Intrathymic deletion of self-specific T cells is substantially AIRE-dependent (47), but the process of selection against tissue-restricted antigens is also a plausible mechanism by which Tregs specific for self-antigen could be selected in thymus and go on to exert their effects in the periphery.

When considering the origin of the requirement for regulation of immune effectors, it is clear that, in the absence of memory and high affinity IgG, the consequences for monotremes of inadvertently making anti-self antibody responses are mild; all their antibodies are low-titer, low affinity, and transiently produced, so one would surmise that the requirement for regulation is limited. Substantial changes in the FoxP3 gene occurred during the evolution of placentation (18), and the relevance of this is further demonstrated by the failure of regulation in placental animals having mutant forms of FoxP3 lacking critical domains (18).

### Evolution of the placenta – a new organ

The major advantage of placentation over oviparous forms of reproduction is that it greatly increases the chances of reproductive success by prolonging the parental protection of the developing offspring, including increasing the chance of surviving infection courtesy of a more mature immune system, initially supported by high affinity maternal IgG antibodies. A recent detailed study of placental fossils combined with comparative genomic data concluded that all modern placentals (Eutherians) are derived from a common placental ancestor that survived the mass extinction 65 Myr ago that eliminated terrestrial dinosaurs (48). Inferences from this study are that this common Eutherian ancestor had a hemochorial placenta with the fetal and maternal blood circulations in intimate contact. Marsupials represent an intermediate step toward this state; they are born very immature and before their immune system develops, but nevertheless get the benefit of protection from predation by *ex utero* occupation of the maternal pouch, where lactation provides the added advantage of maternally transferred antibodies. Marsupials have a yolk sac (Metatherian) placenta, which is simple and relatively impervious to feto-maternal exchange, thus dodging the issue of maternal recognition of fetal and placental antigens. In Eutherian mammals, however, the placenta is fully adapted to cope with a fetus that develops to maturity. There are many new genes that arose during the evolution of placentation to program the development of the placenta (a fetally derived organ) (49), and in addition there are genes essential for survival of the fetus itself *in utero*. The common ancestor of marsupials and placentals, in which these new genes were evolving, had the capacity to make high affinity antibodies; for the hemochorial placenta with its proximity of maternal and fetal circulations, the selective drive for immune regulation capable of protecting the fetus from rejection by its own mother was clearly decisive.

Two facts need to be remembered concerning the placenta:

- Morphologically it is very diverse in different classes of mammal, reflecting the strong evolutionary pressure for mammals with different lifestyles to adapt reproductively to different external conditions. This is reflected in the evolution of new genes and new gene families in different mammalian classes and is particularly evident on the X chromosome, where many of the placental genes are concentrated and which also evolved from an autosomal chromosome in placentals (5).
- Placentation is an intermittent phenomenon, so maintaining T cell tolerance to the rapidly evolving new proteins that are not present in mammalian females post birth, but to which T cell tolerance will be essential if they are to be reproductively successful is a real challenge. From an evolutionary perspective, it is therefore not difficult to understand why the adaptation to placentation might select for mechanisms of dominant tolerance mediated by Tregs, i.e., where Tregs specific for some of the newly evolving placental and fetal-restricted antigens could suppress maternal effector responses against them, whereas other, maybe less abundant or otherwise less conspicuous, neo-antigens would escape without significant Treg reaction. A further point is that this dominant regulation against thymic expressed placental antigens expressed at the feto-maternal interface could also suppress allorecognition of paternal MHC.

### Evolving maternal tolerance to fetus and placenta

Because it seems very likely that the problem of maternal tolerance to fetal and placental proteins had already been solved in the common eutherian placental ancestor, we looked for genetic differences between marsupials and eutherian placental mammals in the genes linked to induction and selection of Tregs in mTECs in thymus. The T cell costimulatory molecule CD28 is vital for Treg selection (50), and CD28 ligands, particularly CD80, are expressed on AIRE^+^ mTECs. In all eutherian placental genomes examined, AIRE, the gene associated with selection of antigen-specific Tregs is chromosomally co-located with the CD28 paralog, ICOS-ligand. The ICOS signaling pathway is crucial for the generation of high affinity antibodies in GCs (51); particularly pertinent to the genetic association between ICOSL and AIRE, it is also required for the effective Treg suppressive function (52). Thus the ICOS gene is a pivot between high affinity antibody production and effective Treg function against immune responses to self, raising the very interesting possibility that the expression of this gene facilitated the selection of self-antigen-specific Tregs found in GCs (43), which then edit self-reactive B cells in GCs driven by ICOS signaling interactions.

The comparative genomics of these interactions is instructive. In all marsupial genomes examined, AIRE and ICOSL are not co-located, but are on separate chromosomes, as is also the case in reptiles and birds. Maybe the genetic translocation that brought AIRE and ICOSL together in the common placental ancestor also mediated the co-expression of AIRE with ICOSL on mTECs. We think that this co-expression was likely to have facilitated development of the process of selecting antigen-specific Tregs, and see it as an illustration of how mutually beneficial processes could serve as selective advantages for one another: here the genetic mechanism for improving antibody affinity (ICOS signaling) co-evolved with the genetic mechanism maintaining tolerance to self (selection of antigen-specific Tregs via AIRE) to modify the generation of high affinity antibodies to tissue-restricted antigens – in this case those expressed in the placenta and fetus. This allows antibodies to pathogens to be transferred to offspring, protecting them from infection without causing autoimmunity, which is a genuine risk, as there are many examples of passive maternal transfer of IgG autoantibodies causing disease in the neonate. In FoxP3^KO^OX40CD30^KO^ mice where not only affinity maturation, but also autoantibody production is impaired, the requirement for Tregs is obviated.

Other studies have linked Tregs specifically with the maintenance of allotolerance at the feto-maternal interface (53–55). However, our data from the FoxP3^KO^OX40CD30^KO^ mice do not support this interpretation. Our data show that FoxP3^KO^OX40CD30^KO^ mice are fully able to reject allografts (data not shown) but we found no evidence that female FoxP3^KO^OX40CD30^KO^ mice rejected allogeneic fetuses. Recent data suggest that immunosuppression in the fetal circulation need not be cell-mediated and implicates the expression of arginase by fetal blood cells (56). Because the fetus is normally sterile, such a global suppression of immune responses in the fetal circulation, and therefore by definition also at the fetal/maternal interface in the placenta, is in our view, a potential mechanism to prevent maternal lymphocytes that enter the fetal circulation inducing rejection. The selective expression of immunosuppressive arginase in the fetal red blood cells in the fetal circulation also helps to explain why lymphocytes in the maternal compartment are still able to respond. For example, although in pregnancy there is susceptibility to some infections, notably influenza, pregnant women make good antibody responses after vaccination (57). We think, therefore that the requirement for regulation in the form of Tregs is more relevant in the context of the mother, to allow antibody responses in particular to foreign proteins derived from infectious pathogens (maternal IgG then passively protects offspring after birth) to go ahead, but to head off responses to fetal or placental antigens that enter the maternal circulation.

## Minimal Immune System for Mammalian Health

Our studies had shown that the TNF-family members, OX40 and CD30-ligand were crucial for the development of high affinity antibodies and memory (38). Although CD30 and its ligand were present in the common amniotic ancestor, true orthologs of OX40-ligand, which plays the dominant role in both affinity maturation and memory, are only present in the mammalian lineage (www.ensembl.org). To ask the question of whether CD4 regulation and memory could have co-evolved in the common placental ancestor we reasoned that mice deficient in FoxP3 (no Tregs) and also deficient in OX40 and CD30 (no high affinity antibodies and memory) would mimic at least to some extent the immune system of the common mammalian ancestral immune system seen in monotremes, and would not exhibit autoimmunity. This was the case (58).

Our mice are interesting in several respects. First, the generation of autoantibodies is abrogated in these mice and they fail to develop the widespread autoimmunity seen in mice and men. The development of FoxP3^KO^ disease is CD4 dependent (19) but the FoxP3^KO^CD30OX40^KO^ mice do not behave as CD4-deficient as they do generate GCs and switched antibody not dissimilar to that observed in monotreme responses. Furthermore, they control many herpes viruses (CD8 immunity is preserved) as well as bacteria. The behavior of the immune responses in the CD30OX40^KO^ mice is mirrored in a single reported case of OX40-deficiency in humans (59). Although the individual did suffer from Kaposi’s sarcoma, she controlled common Herpes virus infections, and was not unduly susceptible to bacterial infections, despite the absence of recall CD4 memory responses.

If one accepts Zinkernagel’s view that the development of memory and high affinity antibodies is a strategy optimized for reproductive success rather than individual survival, then the Foxp3^KO^OX40CD30^KO^ immune system in our view represents the “minimal immune system for health,” the necessary and sufficient platform that had evolved in the common mammalian ancestor before the evolution of high affinity antibodies and memory induced a compromise in the form of susceptibility to autoimmunity.

### Human autoimmunity and blockade of OX40 and CD30 signaling pathways

In human genome wide association studies (GWAS) class II polymorphisms are the strongest genetic link, highlighting the role of CD4 T cells in immunopathology, either due to failure to select antigen-specific Tregs in thymus that subsequently protect, or because CD4 effector T cells drive pathology. The significance of the Foxp3^KO^OX40CD30^KO^ mice with a global defect in Tregs is that it suggests that blocking OX40 alone, or in combination with CD30, would be very effective treatment for wide number of CD4 driven autoimmune diseases without rendering patients susceptible to infection. This is further supported by evidence that shows that FoxP3^KO^ disease can also be blocked by co-injection of antibodies that block OX40 and CD30 signaling pathways (58).

### Cancers mimic the immune evasion strategy of pregnancy

The link between placentation and cancer is hardly new (60). From our perspective, a substantial role for dominant FoxP3-dependent regulation was to facilitate the growth of the fetus and placenta, both expressing many neo-antigens, while at the same time being permissive particularly for the generation of antibodies to pathogens. Recent whole exome sequencing of human cancers has also revealed their enormous heterogeneity (61). Although in theory this should render tumors immunogenic to the CD8 immune system in particular, it is clear that dominant Treg tolerance is a major stumbling block to the development of effective anti-tumor immune responses if Tregs specific for self-antigens can suppress immune responses to tumor-specific antigens.

Two gene examples illustrate the point. The first is the gene alpha-fetoprotein (AFP), the fetal albumin adapted to *in utero* survival, an autosomal gene. The second is the gene PLAC1 expressed in the trophoblast of the placenta of all placental animals, and exclusive to placental animals. Both of these genes are part of the genetic adaption to placental reproduction, but they are also widely expressed in human cancers. This fact suggests that the suppressive effect of fetal and placental antigens on immune responses might have led to the success of cancers that express them. As stated earlier, medullary epithelium in thymus (mTEC) is crucial for the selection of Tregs but not conventional T cells (44). Both PLAC1 and AFP are over-expressed in mTECs [compared to cortical epithelium (cTECs)] (our own data and also www.immgen.org), so it is quite plausible that Tregs specific for these proteins could be selected in thymus. Support for this type of dominant tolerance preventing immune responses to cancer is also provided by the following study (62).

In this study, T cell receptors (TCRs) from tumor infiltrating Tregs found in a murine model of prostate cancer were cloned. TCR transgenic mice positively selected Tregs in thymus in both male and female mice, indicating that they were not tumor-specific Tregs, and as they were found in female mice, were not selected in prostate! Their mTEC thymic derivation was further supported by the observation that selection was dependent on AIRE, the gene that controls expression of many tissue-restricted antigens in the thymus (45).

### De-tuning the immune system to unblock CD8 anti-cancer immune responses

Strategies that suppress Treg function [CTLA4 blockade (63, 64) and PD-1 (65)] have been effective in releasing CD8 anti-tumor immune responses, particularly when used in combination (66). Because Tregs suppress CD4 driven autoimmunity, autoimmunity is a major cause of morbidity and mortality in these treatments. Like Foxp3^KO^ mice, CTLA4^KO^ mice die of CD4 driven autoimmunity (67) so in reality CTLA4 blockade can only be partial in human patients.

However, our studies in FoxP3^KO^OX40CD30^KO^ mice suggest that that CD4 mediated immunity can be obviated in FoxP3^KO^ without seriously compromising autoimmunity. To test whether these mice were capable of mounting anti-tumor immune responses we used the well established murine melanoma line B16 (68). This tumor grows rapidly in syngeneic B16 mice but tumor growth is virtually abrogated in FoxP3^KO^OX40CD30^KO^ mice (our unpublished observations). To us this observation has potential important implications for the treatment of human cancers as it offers the option of permitting effective CD8 anti-tumor responses while preventing the unpleasant CD4 driven autoimmune side effects.

## Summary

In this perspective we outline a strategy for attenuating CD4 driven immunopathology by blockade of the TNF super family members, OX40L (in particular), and CD30L (synergistic with OX40L). Studies of immune function in mice deficient in OX40 and CD30 reveal that although CD4 immunity is reduced, deficient mice are able to deal with the common viral and bacterial infections that can be associated with conventional immunosuppressive strategies. We suggest that antibodies that block these pathways may have therapeutic benefit in human autoimmune diseases mediated by CD4 T cells without compromising resistance to infection.

Recent work has shown that blockade of regulatory T cell function with CTLA4-blocking antibodies has revealed impressive “repressed” CD8 immune responses to neo-antigens expressed by human cancers, particular melanoma, but also some other solid tumors. However, this has been at the expense of CD4 driven autoimmunity that can have considerable morbidity and even mortality. Our work indicates that in the absence of OX40 and CD30, FoxP3-dependent Tregs are dispensable, and mice deficient in OX40, CD30, and FoxP3 mount excellent CD8-dependent anti-tumor immune responses.

As stated above, it is our view that human susceptibility to autoimmunity and cancer are the evolutionarily acceptable side effects of the immune adaptations that evolved in early placental mammals to accommodate a fundamental change in reproductive strategy, and by reversing this process, a detuned and deregulated immune system is much better equipped to mount anti-tumor immune responses against cancers but is also resistant to chronic CD4 driven autoimmune disease.

## Conflict of Interest Statement

The authors declare that the research was conducted in the absence of any commercial or financial relationships that could be construed as a potential conflict of interest.
