# Corrigendum: Evolving Strategies for Cancer and Autoimmunity: Back to the Future

**DOI:** 10.3389/fimmu.2014.00286

**Published:** 2014-06-13

**Authors:** Peter J. L. Lane, Fiona M. McConnell, Fabrina M. Gaspal, Maher G. Nawaf, David R. Withers, Graham Anderson

**Affiliations:** ^1^MRC Centre for immune Regulation, Birmingham Medical School, Birmingham, UK

**Keywords:** CD4 T cell, autoimmunity, tolerance mechanisms, cancer, regulation, memory

The order of the authors for the article “Evolving strategies for cancer and autoimmunity: back to the future” should be as follows: “Peter J. L. Lane, Fiona M. McConnell, Fabrina M. Gaspal, Maher G. Nawaf, David R. Withers, Graham Anderson.”

## Conflict of Interest Statement

The authors declare that the research was conducted in the absence of any commercial or financial relationships that could be construed as a potential conflict of interest.

